# Toward reproducible tumor organoid culture: focusing on primary liver cancer

**DOI:** 10.3389/fimmu.2024.1290504

**Published:** 2024-03-20

**Authors:** Lianming Guo, Chao Li, Weiqiang Gong

**Affiliations:** Department of Hepatobiliary and Pancreatic Surgery, Weifang People’s Hospital, Weifang, Shandong, China

**Keywords:** cancer organoids, primary liver cancer, reproducibility, standardization, tumor heterogeneity

## Abstract

Organoids present substantial potential for pushing forward preclinical research and personalized medicine by accurately recapitulating tissue and tumor heterogeneity *in vitro*. However, the lack of standardized protocols for cancer organoid culture has hindered reproducibility. This paper comprehensively reviews the current challenges associated with cancer organoid culture and highlights recent multidisciplinary advancements in the field with a specific focus on standardizing liver cancer organoid culture. We discuss the non-standardized aspects, including tissue sources, processing techniques, medium formulations, and matrix materials, that contribute to technical variability. Furthermore, we emphasize the need to establish reproducible platforms that accurately preserve the genetic, proteomic, morphological, and pharmacotypic features of the parent tumor. At the end of each section, our focus shifts to organoid culture standardization in primary liver cancer. By addressing these challenges, we can enhance the reproducibility and clinical translation of cancer organoid systems, enabling their potential applications in precision medicine, drug screening, and preclinical research.

## 1 Introduction

Breakthrough in precision cancer therapy requires models that can accurately reflect both inter- and intratumor heterogeneity. Although traditional methods facilitate insights into disease pathology, they are unable to preserve the genetic and phenotypic heterogeneity of original tumor tissues. Both *in vitro* 2D culture and *in vivo* animal models such as patient-derived xenografts and genetically engineered models, present numerous limitations that have been extensively reviewed (1–7). These limitations include inadequate recapitulation of the tumor microenvironment (TME), genetic and phenotypic heterogeneity, drug response, immune response, native interactions, native tissue architecture, and native cellular and extracellular matrix (ECM) components. Additionally, high costs, slow procedures, and ethical concerns of animal models further contribute to the challenges associated with these models. Due to these limitations, conventional preclinical cancer models fail to precisely forecast the clinical efficacy of anticancer therapies, resulting in expensive and time-consuming clinical trials with a significantly low success rate (6, 7).

Organoids are promising models for modeling patient-specific cancer biology. However, their application in (pre-) clinical settings is limited by various factors. One significant obstacle is the low and unpredictable efficiency of organoid derivation and *in vitro* expansion for numerous cancer subtypes (8, 9). Moreover, non-epithelial cancers, such as glioblastoma (10) and rhabdoid tumors (11), have received little attention in organoid research. Furthermore, established organoid cultures usually comprise only neoplastic cancer cells and cannot facilitate long-term co-culture with other cell types in the tumor microenvironment. Finally, the underlying mechanisms of how the extracellular matrix contributes to tumor organoid phenotype and drug sensitivity remain largely unexplored due to the absence of suitable 3D culture platforms to model these interactions (5). The limitations of cancer organoids can be attributed, at least in part, to the utilization of non-standardized and imprecisely defined culture procedures in various studies. Such protocols introduce technical variability into *in vitro* organoid cultures, reducing their capacity to accurately represent the inherent biological heterogeneity of cancer. Technical variability stems from non-standardized cancer tissue sources and their processing, ill-defined and non-specific medium formulations, as well as the use of heterogeneous, animal-derived 3D matrices that lack the necessary tunability to mimic the characteristic of the native tumor ECM (5).

Many studies have reviewed organoid culture strategies in the liver and other tissues (12–14). These strategies include the use of holistic organoid culture approaches, such as air-liquid interface culture, microfluidic 3D culture, and reconstitution approaches, including submerged Matrigel culture and spheroid culture. Bioreactors and bio-printing technology can also be used in combination with the mentioned organoid culture strategies. The utilization of bio-printing technology in organoid culture aims to precisely control cell layering and generate 3D structures. This technology allows researchers to create 3D cultures of single or multiple types of liver cells that can mimicking the complexity of tissues (14). Histological, pharmacological, and multiomics assessments of liver patient-derived organoids (PDOs) show that they effectively retain the diverse features of histological architecture, pharmacotypic properties, gene expression, and genomic landscape from the original tumor (15, 16). As a result, PDOs maintain distinctions between different tumor tissues and subtypes (15, 16). Furthermore, studies have demonstrated that liver organoids can maintain the genomic features and histological properties of their originating tumors even during long-term culturing (approximately 1 year) (16–19). However, as expected, infiltrated immune cells and tumor stromal cells were not propagated in the liver organoids (17). Different organoid culture methods may vary in their ability to preserve specific features of the original tumor. For instance, holistic approaches, as opposed to reconstitution methods, often maintain the histological architecture and immunological components (12). However, in the context of long-term culture, immune cells may not persist. In immunotherapy and immune research studies utilizing reconstitution methods, exogenous immune components are typically introduced to the organoid culture (12). Considering the advantages and limitations of each organoid culture strategy, numerous applications emerge. For instance, liver organoids demonstrate a wide range of applications (20–22), including disease modeling, drug screening and development, precision medicine therapy, immunotherapy, regenerative medicine, and the study of organogenesis and tumorigenesis. For instance, organoids can be employed in disease modeling using CRISPR/Cas9 technology (23). Liver disease or cancer organoid models can be generated directly from diseased donors or, alternatively, from healthy donors by introducing disease-specific mutations into the liver organoids using CRISPR/Cas9. This approach is utilized to investigate mutation-related mechanisms and clinical phenotypes in liver diseases and cancer. Various organoid culture platforms possess distinct properties, which may lead to irreproducibility in study outcomes when different methods are employed. Additionally, the lack of standardization in organoid models exacerbates this issue, contributing to irreproducibility within specific strategies. To enhance the reproducibility of results within these strategies, it is crucial to establish standardized protocols. This involves addressing variations in different aspects of organoid culture that could impact the success rate of organoid generation and study outcomes.

Liver cancer is the second leading cause of cancer-related death worldwide (24), with rising incidence rates linked to risk factors like diabetes and obesity (25, 26). The limited effectiveness of therapeutics and high drug failure rates have led some to consider liver cancer as “not druggable” (27). Intratumor and interpatient genetic heterogeneity may contribute to the lack of targeted agent activity and drug response heterogeneity (28, 29). Organoid culture models provide a means to simulate this heterogeneity, though they come with their own limitations, as discussed above. To tackle issues of irreproducibility and inadequate recapitulation of microenvironment heterogeneity in future studies, it is crucial to implement standardized tools and strategies. In this review, we propose approaches for the standardization of organoid culture to tackle these limitations with a focus on liver cancer organoid culture standardization. Indeed, we present a critical framework that addresses these concerns by discussing the limitations of current protocols and recent innovations that have been developed to mitigate them.

## 2 Source, collecting methods, and subsequent processing of tumor tissue

There has been considerable variation in the techniques employed for the generation of organoids, which encompasses the selection of tumor tissue sources and their subsequent processing. For instance, organoids have been generated from primary tumors (30–34), metastatic lesions (35–38), circulating tumor cells (39–41), and tumor cells obtained from liquid effusions (42) such as pleural effusion (43). Various techniques, such as solid and liquid biopsies, surgical resections, rapid autopsies (44), and scratching the mucosal membrane (45, 46), are used for obtaining samples. Following the collection of tumor tissue, patient samples need to be processed for subsequent culture, which often involves encapsulation in a 3D matrix. Multiple strategies have been developed for the culture of tumor organoids, but two predominant approaches have emerged (12).

The first strategy involves reconstituted models where organoids consisting solely of cancer cells are generated. In this model, fresh tumor tissue samples are cut into small pieces, washed with ice-cold PBS, and subsequently enzymatically digested with vigorous pipetting. The supernatant is collected and centrifuged, then the cell pellet is suspended in Matrigel and dispensed into culture plates. Tissue dissociation in organoid preparation activates ROCK-dependent programmed cell death. The addition of ROCK inhibitors to the medium is known to effectively increase organoid generation success rates, as supported by various studies (47, 48).

The second approach involves holistic models, where native TME models preserve the intrinsic immune microenvironment of tumor specimens along with tumor cells without any reconstitution. In this approach for organoid generation, which includes air-liquid interface (ALI) culture and microfluidic culture, minced primary tissue fragments are mixed with collagen. In microfluidic culture, fresh tumor samples were initially placed in media (either DMEM or RPMI) on ice and then finely chopped in a standard 10cm dish using sterile forceps and a scalpel. These chopped tumor pieces were enzymatically digested, and after digestion, the resulting minced tumor samples were suspended in fresh media. The suspension was then filtered sequentially through 100 μm and 40 μm filters to obtain three different fractions: S1 (>100 μm), S2 (40–100 μm), and S3 (<40 μm) spheroid fractions. The 40‐100 μm-sized spheroid fractions collected were then transferred to ultra-low attachment tissue culture plates. These spheroid fractions were mixed with collagen, and the resulting spheroid-collagen mixture was introduced into the 3D microfluidic chamber (49). In ALI approach, fresh tissue samples are firstly washed in ice-cold culture medium or PBS to eliminate additional contents such as blood. Subsequently, the tissue is transferred to a hood, rinsed multiple times in ice-cold medium or PBS, and minced thoroughly with iris scissors on a sterile surface such as a tissue culture plate lid. The mincing process aims to achieve tissue fragments approximately 0.3 mm³ or smaller with a viscous and nearly homogeneous appearance, ensuring suitability for culture. This mincing process is usually completed within 5 minutes on ice to prevent cell damage and tissue drying (50). Obtained tissue fragments are cultured in a mixture with collagen gel on the top layer of an inner transwell dish. Nutrient-rich culture medium permeates from an outer dish through a semi-permeable membrane into the inner dish. The upper surface of the organoid culture is in direct contact with the air, facilitating cells’ access to a sufficient oxygen supply (51).

**Figure 1:**
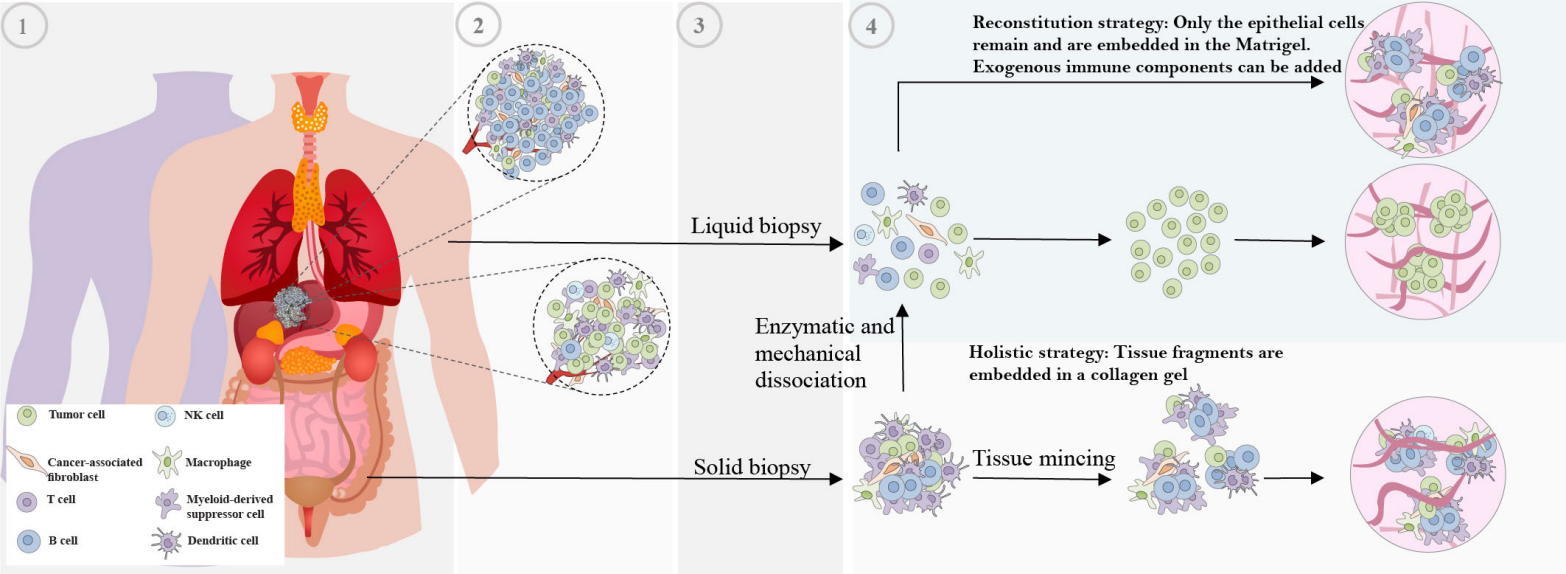
Non-standardized methods of tissue obtaining and subsequent processing for patient-derived organoid generation. The various technical variability factors contribute to the lack of standardization in patient-derived organoid generation. These factors include: 1 and 2) Interpatient and intratumor heterogeneity, 3) Variation in obtaining tumor tissue techniques, and 4) Variation in organoid generation processing protocols.

The current implementation of these strategies is not standardized, which impairs their valuable contributions to clinical research. By reproducible advancements that are focused on eliminating technical variability to achieve more accurate organoid models that better reflect the complexity of cancer, as illustrated in **Table 1**; **Figure 1**.

**Table 1:** Limitations and potential solutions of cancer-tissue sources and processing in tumor organoid culture.

Limitations	Solutions	Refs
**Limited representation of tumor heterogeneity.**	Obtain tissue samples from multiple regions of the tumor to capture its spatial heterogeneity and temporal changes.	(42, 52, 53)
**Clinical characteristics affecting organoid generation.**	Consider clinical factors such as cell-type heterogeneity, cancer subtype, tumor recurrence, histopathological grade, and patient treatment status when generating organoids.	(16, 42, 54, 55)
**Biased representation of patient populations:** Biobanks may not include diverse patient populations, leading to potential biases in drug discovery and biomarker development.	Ensure a diverse representation of patient populations in biobanks to avoid skewed results and improve generalizability. Include samples from different demographics and treatment histories.	(5, 16, 42, 54, 55)
**Contamination by healthy cells.**	Carefully assess tumor cell purity to avoid contamination by rapidly growing healthy cells. Develop methods to selectively isolate neoplastic cells and minimize interference from healthy tissue.	(8)
**Variability in tissue processing:** Inconsistencies in tissue dissociation methods and fragment sizes can affect organoid generation and reproducibility.	- Develop standardized dissociation conditions for different tissue types to minimize non-specific cleavage of cell-surface proteins. - Optimize mechanical and enzymatic dissociation techniques for reproducibility. - Explore techniques such as microfabrication or microfluidics to control cluster size and uniformity.	(56–59)
**Limited control over defined cell populations:** Mincing or embedding intact tumor fragments may result in non-uniform microenvironments and lack control over encapsulated cell populations.	Explore microfabrication and microfluidic technologies to improve tissue fragment uniformity and enhance control over cell composition in organoid cultures.	(56, 58, 59)
**Disruption of native cell interactions:** Complete tissue dissociation disrupts complex cell-extracellular matrix interactions and removes non-neoplastic or non-epithelial cell types.	Explore alternative approaches, such as mincing intact tumor fragments, to preserve native tissue architecture and the tumor microenvironment.	(9, 59)
**Lack of standardized clinical tissue collection:** Clinical requirements and timing of tissue collection can vary, impacting organoid generation.	- Collaborate with clinicians to optimize tumor tissue collection methods. - Explore techniques such as using frozen tissue samples to overcome issues related to the timing and availability of fresh samples.	(5, 56, 60)
**Incorporation of tumor microenvironment heterogeneity.**	- Develop culture platforms that mimic the complexity of the TME, incorporating CAFs, immune cell populations, and relevant cell-cell interactions. - Utilize scaffold-based or biomimetic culture systems that mimic the extracellular matrix composition and stiffness found in the native tumor microenvironment.	(12, 61–63)
**Limited long-term culture capabilities:** Some organoid culture methods may have limitations in maintaining organoids over extended periods.	Explore modifications to culture techniques, such as altering embryoid body size and shape or using slice culture, to enhance the long-term culture of organoids.	(64)
**Time-consuming organoid development:** Traditional organoid culture methods can take several weeks to develop, delaying research and testing.	Develop rapid and reliable methods, such as acoustofluidic assembly, to accelerate the generation and evaluation of organoids for various applications.	(56, 65)

### 2.1 Tumor tissue source

In order to accurately model the biological heterogeneity of cancer in individual patients using organoids, it is necessary to obtain tissue samples that reflect the spatiotemporal diversity of the tumor. However, the currently available cancer organoid models are mainly derived from single biopsies or small fragments of surgically resected tissue, and thus do not adequately represent the cellular and morphological diversity or the temporal evolution of the parent tumor *in vivo*. Despite the high efficiency of generating cancer organoids from certain cancer subtypes, various clinical characteristics such as intra-tumoral cell-type heterogeneity, cancer subtype, histopathological grade, tumor recurrence, and patient treatment status can significantly affect the ease of generating organoids from cancer tissues (16, 42, 54, 55). Indeed, treatment before tissue collection could affect the phenotype of organoids and their responses to drugs *in vitro*. Moreover, biased representation of patient populations may result in misguided drug discovery and biomarker development if the response to anticancer treatments differs in populations absent from biobanks.

The presence of rapidly growing healthy cells in tumor tissue samples can contaminate studies aimed at exclusively recapitulating neoplastic cell biology (8), highlighting the need for careful assessment of tumor cell purity. The overgrowth of healthy tissues contaminants in the liver and other tumor organoids has been observed (8, 16, 30, 66–70), possibly attributed to a higher occurrence of mitotic failure and subsequent cell death in tumor cells (71). Removing this contaminant to obtain pure tumor organoids is crucial for increasing the success rate of organoid generation, the reliability and reproducibility of research findings, the translational potential and precision medicine, and understanding tumor-specific characteristics, heterogeneity, and establishing representative models (8, 16, 30, 66–70). There are several ways to avoid the growth of normal cell contamination in tumor organoids, and one of them is using selective pressure (30, 67, 70). This involves adjusting the composition of the culture medium by either removing specific growth factors essential for the growth of normal cells or adding inhibitors to selectively support the growth of tumor cells. However, this approach does not result in tumoroid generation from all tumor samples compared to the classical medium. For instance, in liver cancer, the yield of cancer organoids was improved by removing Rspo-1, Noggin, and Wnt3a from the medium and adding dexamethasone and a ROCK inhibitor (16). The second approach involves physically removing healthy cell contamination based on organoid structures, organoid morphology, and histology (16, 67, 69, 70). In the third method, the digestion time is increased to avoid the growth of non-tumoral contaminants in liver organoids, resulting in a reduced yield of non-tumoral contaminants (16).

The tissue method collection also can significantly affect organoid generation, but researchers have no control over them. The time interval between the collection of patient tissue and subsequent 3D encapsulation also varies across protocols and could considerably affect the efficiency of organoid generation (5).

#### 2.1.1 Standardization of tumor heterogeneity

Modeling the tumor heterogeneity is one of the challenges in the standardization of organoid culture. Studies showed that single-region tissue samples do not adequately represent the extensive spatial heterogeneity of cancer clones, and sampling of multiple regions of the tissue will facilitate the development of more accurate tumor organoid models. For instance, Roerink et al. generate clonal organoids from single cells sourced from four to six distinct tumor tissue sections of three untreated colorectal cancer patients (52). Analysis of each CRC organoid culture in the same culture condition exhibited clone-specific epigenetic and transcriptomic features along with diverse drug response patterns. Indeed, multi-region cancer organoid models capture intratumor, transcriptomic, morphological, and pharmacotypic heterogeneity (42). Song et al. isolated 15 single clones from four tumor organoid lines from one patient to study heterogeneity within a single tumor tissue (53). Each organoid line showed distinct alterations in genotype and phenotype. They observed differences in mutational patterns and drug responses, including time-lapse responsiveness (53).

Some studies have used the addition of exogenous cell components to organoid cultures for modeling tumor heterogeneity (12). For instance, Öhlund et al. employed a coculture system to study the interaction between pancreatic ductal adenocarcinoma organoids and cancer-associated fibroblasts (CAFs) (61). Intriguingly, RNA sequencing analysis revealed the existence of two distinct subpopulations of CAFs, characterized by a myofibroblastic or immunoinflammatory phenotype, and demonstrated their unique interactions with cocultured pancreatic ductal adenocarcinoma organoids. Similarly, Ebbing et al. employed patient-derived CAFs in the coculture of esophageal adenocarcinoma organoids and found that interleukin-6 produced by stromal cells was responsible for driving epithelial-to-mesenchymal transition and therapeutic resistance. Consequently, novel biomarkers and therapeutic strategies for patient stratification and personalized treatment were proposed (72). Importantly, murine CAFs from the same patient-derived xenografts did not exhibit the same phenotype, emphasizing the limitations of murine models in replicating the human TME.

In immunological studies involving organoids, it is essential to uphold the heterogeneity of the tumor immune microenvironment by preserving the components of immune cells and intricate cellular interactions. To attain this objective, Neal, Li, et al. employed the advantages of the ALI culture system to generate tumor organoids that can recapitulate tumor immune microenvironment. These organoids retained native tissue architecture, CAFs and immune cell populations, facilitating personalized immunotherapy tests (9). The study validated the possibility of replicating the *in vivo* tumor-infiltrating T-cell repertoire and simulating patient-specific PD1/PDL1-dependent mechanisms of immune suppression. In a separate investigation, researchers created an innovative culture platform to assess the efficacy of cancer immunotherapies on organoids derived from patients with colorectal cancer (CRC). This involved the utilization of human chimeric antigen receptor-engineered natural killer (CAR-NK) cells (62). By employing live cell imaging, they successfully monitored the recruitment of natural killer cells and observed antigen-specific cytotoxicity against individual organoids expressing diverse cancer-relevant targets.

##### 2.1.1.1 Standardization in primary liver cancer

A limited number of studies have attempted to model tumor heterogeneity in primary liver cancer. For instance, Li et al. (28) were the pioneers in identifying interpatient and intratumor functional (drug response) heterogeneity in primary liver cancer. To achieve this, they established multi-region organoid cultures from primary human liver cancer, generating a total of 27 PDO cultures from 5 human primary liver cancers. In a recent study, Ji et al. (15) developed a patient-derived liver cancer organoid biobank (LICOB). LICOB effectively captures the histological and molecular properties of various liver cancer types, as evidenced by comprehensive multi-omics profiling. Proteogenomic profiling of LICOB identified two distinct organoid subtypes, namely the proliferative and metabolic subtypes, which are correlated with patient prognosis. Notably, high-throughput drug screening showed that each subtype displays a unique response pattern, which is intricately linked to specific multiomics signatures.

#### 2.1.2 Standardization of tissue collection timing

Certain limitations of organoid culture may be beyond the control of researchers. For example, an issue is related to the timing of tissue collection and the availability of fresh samples. To overcome this challenge, Walsh et al. (60), have developed a technique to generate viable cancer organoids from frozen primary human breast cancer tissue after 6-12 months of storage. The drug response profile of the mentioned organoids was comparable to that of fresh organoid cultures obtained from the same biopsy at the time of collection. Wu et al. (56) present a novel approach for rapid and reliable evaluation of cancer immunotherapy using primary tumor-derived organotypic cell clusters (POCCs). The researchers utilized a label-free, contactless, and highly biocompatible acoustofluidic method to assemble hundreds of cell clusters from patient primary breast tumor dissociation within a short time frame of two minutes. The POCCs were established and evaluated within 12 hours, which is much faster than the current tumor organoids that usually take more than two weeks to develop. The researchers also incorporated time-lapse living cell imaging to ensure faithful recapitulation of cancer-immune interaction dynamics and their response to checkpoint inhibitors. The POCCs demonstrated a superior ability to preserve the cell components from the primary tumor due to the short culture time (56).

##### 2.1.2.1 Standardization in primary liver cancer

He and colleagues (73) conducted a study to determine the feasibility of using a cryopreservation technique to preserve tumor tissue specimens from liver, lung, renal, thyroid, and colon cancers for 3D organoid cultures. Following surgical resection, they minced and immersed the specimens in CryoStor™ media. Subsequently, the specimens were gradually frozen at a rate of −1°C per minute at −80°C for 24 hours and then stored in liquid nitrogen. After 15–18 months, the tissues were thawed and converted into single-cell suspensions, which were then assessed for cell viability. The study found that the tissues could be successfully frozen and thawed multiple times without significantly impacting the viability and growth of the 3D organoid culture. Based on these results, the authors concluded that the cryopreservation technique could be used to standardize the preservation of tumor tissue specimens for 3D tissue cultures, thereby improving the accessibility of viable human tumor tissue/cells in a time-independent manner.

### 2.2 Tissue processing for organoid generation

Inconsistencies in the processing of tumor tissue also contribute to the lack of standardization in cancer organoid cultures. In a commonly used approach, patient-derived tissue is dissociated into single cells through mechanical and/or enzymatic means, and subsequently embedded in a 3D matrix submerged in a nutrient-rich medium (57). Complete dissociation of tissue samples facilitates the expansion of clonal organoids, which may be advantageous or disadvantageous depending on the context. However, enzymatic dissociation can result in non-specific cleavage of cell-surface proteins and requires specific dissociation conditions for each tissue type (57). Additionally, tissue dissociation methods generate cell clusters of varying sizes, ranging from individual cells to clusters approximately 100 μm in diameter, which are not consistently reproducible. Lastly, complete tissue dissociation removes the native neoplastic cell interactions with the tumor microenvironment, resulting in the disruption of complex cell-extracellular matrix interactions and negative selection against non-neoplastic or non-epithelial cell types. An alternative approach involves mincing patient tissues, followed by the encapsulation of intact tumor fragments in a 3D matrix. Unlike complete tissue dissociation, this method allows the preservation of native tissue architecture and the TME cell components that could influence organoid formation and phenotype. However, similar to the technique discussed earlier, manual tissue mincing may result in non-reproducible fragment sizes, leading to non-uniform microenvironments for encapsulated cells. This can affect factors such as the flow of oxygen and nutrient gradients throughout large tissue fragments. Moreover, mechanical mincing of tissue may cause damage, reducing the number of viable cells available for organoid generation. Additionally, although intact tumor fragments maintain the native tumor architecture, their use offers limited control over the encapsulation of defined cell populations and their interactions with the 3D environment.

#### 2.2.1 Standardization of fragment uniformity

Recently, some approaches, such as microfabrication and microfluidic technologies, have been utilized to standardize the downstream processes of tissue processing, fragment uniformity, organoid derivation, and pharmacological testing. For instance, a microfabrication approach was developed to generate U-shaped microwell arrays to allow the formation of healthy gastrointestinal and colorectal cancer (CRC) organoids with a predetermined number of initial cells in a reproducible manner (58). By employing automated imaging techniques for organoids grown in microwell arrays, they showed increased homogeneity in size and morphology. In another study, researchers generated cuboidal-shaped sections of human glioma xenograft tumors with submillimeter dimensions, called “cuboids”, which offer improved tissue fragment uniformity compared to traditional tissue mincing techniques (59). Wu et al. established acoustically assembled POCCs with uniform fabricate size and cell composition that has the capability to maintain all the elements of TME found in the original tumors. As a result, it allows for the efficient assessment of the effectiveness of immunotherapies within a brief time (12 hours) (56). Giandomenico et al. introduced an innovative approach that involves altering the size and shape of the embryoid body to enhance its surface area. They utilized slice culture techniques to ensure sufficient nutrient and oxygen supply to the internal regions of the organoid. These strategic modifications have enabled the long-term culture of organoids (64). Gong and colleagues have created an acoustic droplet-based platform to rapidly generate tumor organoids that maintain the original tumor immune microenvironment (65). This platform, combined with a hydrophobic substrate, produces a large number of uniform and viable bladder tumor organoids *in vitro* within a week. These organoids contain all components of bladder tumors, including diverse immune elements and tumor cells. The proposed acoustic droplet platform is an easy-to-use, repeatable, and stable technique, making it a dependable option for personalized tumor immunotherapy.

##### 2.2.1.1 Standardization in primary liver cancer

Jiang et al. (74) developed a high-throughput automated organoid platform to address the issue of non-uniformity in organoid size and cellular components. The platform contains a droplet-based microfluidics system where uniform cell-laden Matrigel droplets are first produced and then precisely arranged into patterns on 96-well plates using a 3D droplet printer. This innovative approach enables the production of organoids larger than 400 μm within only one week, exhibiting both interpatient heterogeneity and interorganoid homogeneity. Furthermore, these organoids accurately mimic 97% of gene mutations found in the original tumor and can be used to predict drug responses. In another study, Takebe et al. (75) developed an omni-well-array culture platform that allows for the mass production of homogeneous and miniaturized liver organoids from human iPSCs. Similarly, Xu et al. (76) designed a micropatterning technique that can be used to generate liver organoids with a uniform deterministic size in a multi-well plate in a reproducible and high-throughput manner. Tienderen et al. (77) developed a one-step liver extracellular matrix-containing micro-encapsulation platform that supports the scalable and size-standardized generation of liver organoids.

## 3 Medium of organoid culture: cost and reproducibility

The development of organoid models to study healthy and diseased human tissue has been facilitated by a deep understanding of the *in vivo* stem cell niche and the regulatory factors that support adult stem cell growth *in vitro*, such as Wnt/R-spondin, Noggin, and epidermal growth factor. Various growth factors and compositions are employed in liver organoid culture, including B27, EGF, FGF10, HGF, [Leu15]-Gastrin I (human), forskolin, ROCK Inhibitor, Noggin, Rspo-1, and Wnt3a (16–18). However, the utilization of each growth factor varies based on the specific aims of studies and the type of liver tissue sample being cultured—whether it is an unhealthy liver state such as nonalcoholic steatohepatitis (NASH) (78), healthy liver, tumor, or a specific tumor subtype organoid. For instance, in their study, Broutier et al. (2017) (16) utilized specific culture conditions to cultivate healthy liver organoids. This involved a medium composition that included Rspo-1, Wnt3a, and Noggin. In contrast, when developing tumoroids, the researchers intentionally excluded these components to minimize contamination with healthy cells. Instead, they introduced dexamethasone into the medium (16). To ensure a comprehensive representation, Broutier et al. employed a dual approach. Half of the samples were cultured in a medium enriched with Rspo-1, Wnt3a, and Noggin, while the other half was subjected to the tumoroid-specific medium. This strategy aimed to facilitate the growth of diverse cancer subtypes. For example, Rspo-1 was identified as crucial for some cholangiocarcinoma patient-derived organoids (16). These distinct formulations allowed for effective disease modeling and drug screening, thereby enhancing the relevance of organoid studies in liver cancer research. However, it is noteworthy that the use of this distinct composition may result in the lack of organoid generation in some tumor subtypes.

**Figure 2:**
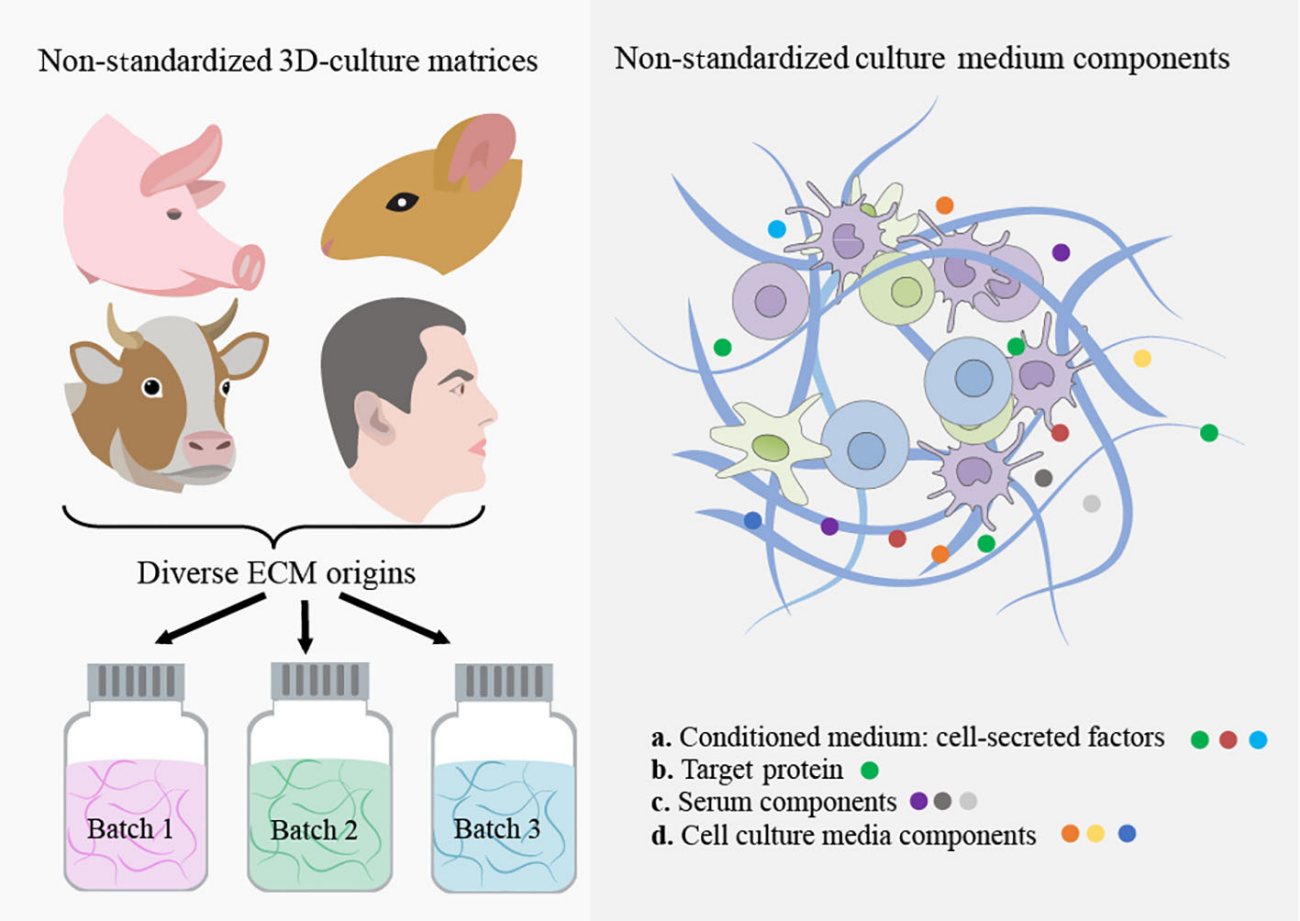
Non-standardized 3D culture matrices and culture-medium components. The use of animal-derived ECMs for organoid culture faces significant challenges due to batch-to-batch variability and the risk of contamination from xenogenic sources. Moreover, these matrices possess poor tunability and intricate, undefined compositions, resulting in limited control over their properties. This ultimately hampers the use of organoids in the cancer research era. The utilization of ill-defined and heterogeneous conditioned medium and serum from animals in organoid culture causes unpredictable effects on organoid phenotype.

In addition to directly adding these proteins to the organoid culture, customized medium formulations are required for each intra and interpatient sample, including cell proliferation and differentiation promotion factors, as well as tumor genotype-specific soluble factors (55, 79–81). Notably, some tumor organoid cultures were found to not require certain components of the medium that were essential for the growth of healthy organoids (79). Unfortunately, current medium components, including purified growth factors, animal-derived serum, and conditioned medium, are either prohibitively costly, difficult to reproduce, or highly variable, making it difficult to precisely model the TME for individual patients. Here, we discuss these challenges in-depth and explore recent developments in creating scalable and standardized cancer organoid medium formulations, as illustrated in **Table 2**; **Figure 2**.

**Table 2:** Limitations and potential solutions of medium formulations in tumor organoid culture.

Limitations	Solutions	Refs
Medium formulations (Genotype-specific)	Develop customized medium formulations for individual patient samples based on their genetic and phenotypic heterogeneity.	(55, 80, 81)
High cost of commercial growth factors	- Use conditioned medium derived from engineered mammalian cells to generate Wnt3a, Noggin, and/or R-spondin, which significantly lowers the cost and enhances accessibility. - Develop recombinant proteins with improved stability and activity, such as using phospholipids and cholesterol as carriers for Wnt3a. - Explore alternative expression systems, such as bacterial expression systems with proper folding techniques, to reduce costs and ensure protein quality. - Develop cost-effective surrogate agonists with similar biological activities as recombinant proteins for medium formulations.	(54, 80, 82–88)
Presence of animal-derived serum in conditioned medium, organoid derivation, passaging, and cryopreservation: Animal-derived serum used in medium formulations has batch-to-batch and supplier-to-supplier variability.	- Reduce reliance on animal-derived serum by using a conditioned medium without serum. - Develop defined and standardized medium formulations that eliminate the need for animal-derived serum. - Develop serum-free medium formulations to eliminate variability and contamination.	(80, 83, 88–90)
Inadequate solubility and stability of recombinant proteins (growth factors)	Use stabilizing factors such as afamin or phospholipids and cholesterol as carriers to enhance solubility and stability.	(80, 82, 83, 88)
Variability in protein activity levels	Avoiding direct dilution of conditioned medium and optimizing purification methods.	(82, 83, 87)
Limitations of bacterial expression systems (Recombinant growth factors)	Explore alternative expression systems with better protein folding and post-translational modification capabilities.	(87)
Immunological studies limitations and infection risk: Animal-derived sera limit human-specific immunological research and increase the risk of bacterial, viral, or zoonotic infections.	Serum-free protein production: Develop serum-free methods for recombinant protein production.	(91)

### 3.1 Cost and reproducibility challenges of growth factors

Since most cancer organoid models are composed of a homogenous tumor cells population, external signaling factors are required to promote cancer cell growth, which are often secreted by TME cells *in vivo*. However, the incorporation of certain components as purified recombinant proteins may be limited due to inadequate solubility and stability for prolonged storage (82), leading to reduced protein activity (83). In addition, the cost of medium formulations containing multiple growth factors and nutrients for large-scale applications can be limiting. Nevertheless, numerous labs have developed a solution to this problem by using conditioned medium derived from mammalian cells that are engineered to generate Wnt3a, Noggin, and/or R-spondin. This solution has significantly lowered the cost and enhanced the accessibility of implementing cancer organoid models in various tissue types (82). But, directly diluting conditioned medium into organoid medium formulations suffers from batch-to-batch variability, leading to fluctuating target-protein activity levels and irreproducibility. Moreover, conditioned medium comprises diverse factors beyond the target protein(s), and their effects on the phenotype and drug response can be unpredictable. The presence of residual serum, usually fetal bovine serum (FBS), in conditioned medium further complicates the situation.

#### 3.1.1 Standardization of growth factor costs

Numerous cost-effective engineered agonists with comparable biological activities have been developed as viable alternatives to the mentioned full recombinant growth factors. For instance, Janda et al. utilized *de novo* design and protein engineering strategies to develop water-soluble, surrogate Wnt agonists that induce Frizzled–LRP5/6 heterodimerization and imitate downstream beta-catenin signaling (84). They further expanded this toolkit by developing next-generation surrogate (NGS) Wnts. Using the TOP-Flash Assay, they demonstrated that NGS Wnts induce similar levels of downstream Wnt signaling at a lower concentration than the earlier generation (85). The use of agonists has been shown to improve the efficiency of generating healthy human organoids *in vitro*, compared to Wnt3a conditioned medium. Besides Wnt agonists, a similar method was employed to design R-spondin surrogates. These surrogates are capable of binding and preventing the degradation of Frizzled and the associated LRP5/6 receptors without the requirement of LGR proteins, which are usually involved in the natural interaction process (86). Hansen and colleagues developed a library of cystine-knot peptides (CKPs) with multiple loops for randomization and selection using directed evolution (92). They discovered picomolar affinity CKP agonists of the human LRP6 receptor, which mimic the natural Wnt inhibitors DKK1 and SOST by binding at the first β-propeller domain of LRP6. However, the CKP agonists differ from natural Wnt inhibitors by amplifying the signaling of natural Wnt ligands rather than activating the pathway on their own. In an organoid model, the CKP agonists induced stem cell activity and stimulated growth in human intestinal organoids (92). This approach could advance the design of next-generation agonist ligands and be applied to other signaling pathways.

Bacterial expression systems also serve as a cost-effective strategy for generating growth factors. However, they have inherent limitations, particularly in the context of protein folding and post-translational modifications. Urbischek et al. introduced a novel approach to express and purify R-spondin 1 and Gremlin 1 in Escherichia coli, addressing the challenges of maintaining their proper folding and configuration of disulfide linkages (87). To achieve proper folding and configuration of disulfide linkages, they co-expressed the disulfide-bond C isomerase with the target proteins in E. coli, which was followed by *in vitro* disulfide shuffling. The efficacy of these recombinant proteins in supporting healthy human colon epithelium and colon adenocarcinoma organoids was comparable to commercially available proteins. The cost of producing R-spondin 1 and Gremlin 1 proteins was reduced significantly compared to commercial sources (87).

##### 3.1.1.1 Standardization in primary liver cancer

As previously mentioned, Miao et al. (85) developed an NGS Wnt that can be used for liver organoids. Fzd subtype-specific NGS Wnt can activate specific Fzd receptors, preventing the non-essential activation of unwanted Fzd receptor types. The use of Fzd subtype-specific NGS Wnts allows for the investigation of individual Fzd subtype receptor functions. Chen and colleagues (93) established a method to create a potent and specific WNT surrogate. They highlighted that achieving strong WNT/beta-catenin activation necessitates the simultaneous binding of this surrogate to multiple FZDs and LRPs. The study demonstrated that effective signaling occurs when two distinct FZDs are recruited alongside LRP. An effective method for triggering the Wnt signaling pathway involves the utilization of inhibitors targeting glycogen synthase kinase 3 (GSK3). This approach has the potential to be both scalable and cost-effective, rendering it suitable for large-scale liver organoid generation. Huang et al. (94) demonstrated that GSK3 inhibitors led to an upregulation of specific gene expressions, aligning with the activation of the Wnt/β-catenin signaling pathway. Torizal and colleagues (95) designed a straightforward technique involving dialysis-mediated medium conditioning, effectively exploiting the buildup of growth factors to enhance the creation of liver organoids derived from human-induced pluripotent stem cells at a substantial cell density. Through the implementation of this uncomplicated, downsized dialysis culture system, they were able to showcase the practicality of achieving cost-efficient, high-density hepatic differentiation with minimal utilization of growth factors. The compact dialysis-based culture system showed the practicality of producing liver organoids in a cost-effective manner, while minimizing the utilization of growth factors. Heidariyan et al. (96) proposed using gelatin-coated polymeric microparticles loaded with growth factors incorporated into 3D spheroids. This innovative approach allows efficient growth factor delivery, leading to comparable hepatocytic marker expression with 10 times fewer growth factors. Wang et al. (97) successfully generated the *in vitro* growth of mouse liver organoids by utilizing a trio of small-molecule compounds. Using small-molecule compounds instead of growth factors can decrease the cost of organoid generation.

### 3.2 The ill-defined and variability challenges of animal-derived serum

Animal-derived sera lack well-defined components and include (xenogeneic) elements, leading to unpredictable impacts on cancer organoid growth and phenotype. Consequently, it hinders human-specific immunological research and raises the risk of bacterial, viral, or zoonotic infections (91). FBS is widely utilized in complete cancer organoid medium formulations, either indirectly through a conditioned medium or directly for organoid derivation, passaging protocols, and organoid cryopreservation. FBS contains a variety of soluble and signaling factors, including hormones, peptides, full-length proteins, lipids, carbohydrates, and several small-molecule nutrients that are known to promote *in vitro* cell culture (89). Studies have revealed the presence of 1,800 unique gene products in human serum (90). This complexity is further complicated by batch-to-batch and supplier-to-supplier variability, which can be attributed to the animal source and differences in serum collection based on geography and seasonality.

#### 3.2.1 Standardization of ill-defined medium formulation

Generally, the reliance on animal-derived serum and conditioned medium in cancer organoid culture hinders the establishment of standardized models and restricts meaningful comparisons of experimental results across various laboratories. For instance, to become biologically active, the lipidated Wnt proteins require solubilization, which is typically facilitated by a serum glycoprotein called afamin/α-albumin supplied through a serum-containing medium (82, 88). Mihara et al. (88) developed a co-expression system utilizing mammalian cells transfected with both Wnt- and afamin-encoding vectors. This system enables the production of solubilized Wnt protein without the requirement of adding serum. Seino et al. showed that substituting serum-stabilized Wnt3A-conditioned medium with serum-free afamin-stabilized Wnt3A enabled the stable culture of organoids for more than 8 months (80). Despite mentioned progress, the use of FBS remains common during the initial expansion of mammalian cells before conditioned medium collection, which can lead to contamination with serum-derived factors and impact protein production variability across batches. Tüysüz et al. (83) have developed a Wnt3a stabilizing method using phospholipids and cholesterol as carriers, that enhance the stability and activity of recombinant Wnt3a. This modification also simplifies its purification from complex conditioned medium contents using Blue Sepharose affinity and gel filtration chromatography, resulting in the recovery of up to 80% pure Wnt3a. This approach, in contrast to the detergent-based solubilization method, significantly increases self-renewal of organ and embryonic stem cells, enabling the establishment of human organoids in serum-free conditions with similar efficacy as serum-containing Wnt3a conditioned medium.

##### 3.2.1.1 Standardization in primary liver cancer

Sekine et al. (98) developed chemically defined, animal origin-free (CD-AOF) media to enable large-scale generation of iPSC-derived liver organoids, guaranteeing culture system quality and reproducibility by reducing lot-to-lot variations and contamination risks. The resultant organoids exhibited comparable hepatic functionality to those cultured with conventional media. In a separate investigation, Wang et al. (99) developed a novel technique to generate human ESCs-derived, expandable hepatic organoids (hEHOs), utilizing entirely defined media (serum-free, feeder-free). These hEHOs effectively retain the characteristics of bipotential liver stem/progenitor cells, preserving the ability to mature into functional hepatocytes or cholangiocytes. The method permits hEHO expansion for 20 passages, facilitating substantial growth for industrial or clinical applications. As previously discussed, Tüysüz et al. (83) demonstrated the successful serum-free generation of human organoids from healthy and diseased intestines and livers using stabilized Wnt3a. To evaluate their stability, they incubated the Wnt3a protein for various durations in the culture medium at 37 °C and assayed the remaining activity using a luciferase reporter assay. They addressed the issue of the limited effectiveness of purified Wnt3a protein due to its rapid loss of activity within culture media. Considering recent advancements, we anticipate that in the future, it will be possible to utilize fully defined culture environments and conditions in organoid cultures.

## 4 Three-dimensional matrices

Tumors exhibit abnormal remodeling of the ECM, affecting its composition, architecture, and mechanical properties (100–102). Altered ECM can impact the biology of neoplastic and healthy cells of TME through biochemical and biophysical interactions. These alterations have been linked to cancer cell behavior, disease progression, metastasis, and drug response (101, 103). Significant progress in 3D tissue culture techniques has revolutionized our understanding of how neoplastic cell behavior is greatly influenced by cell-ECM interactions and 3D tissue organization. For instance, studies have shown that the transcriptional profile of human breast cancer cells is significantly influenced by culture dimensionality and 3D cell morphology (104). Despite the critical role of ECM properties in cancer behavior, there has been limited comprehensive research on the effects of intra- or intertumoral ECM heterogeneity (specific ECM characteristics) on the pathogenesis and response to anti-cancer treatments of patient-derived cancer organoids. Indeed, the scarcity of studies focusing on the standardization of organoid-ECM interactions and the development of reproducible ECMs, alongside the prevalent use of undefined and poorly controllable animal-derived scaffolds in most 3D *in vitro* cancer organoid experiments, has led to irreproducible results in many studies. In this regard, we highlight the limitations of the most commonly used matrices such as EHS matrix and collagen and proposes the use of advanced engineered matrices that provide reproducible control over both biochemical and biophysical ECM properties for cancer organoid culture, as illustrated in **Table 3**; **Figure 2**.

**Table 3:** Limitations and potential solutions of 3D matrices in tumor organoid culture.

Limitations	Solutions	Refs
Batch-to-batch variation in EHS and Collagen matrices	Develop synthetic matrices	(5, 105–107)
Ill-defined, inconsistent protein content and xenogenic contaminants in EHS and Collagen matrices	Purify the matrices to remove contaminants or develop synthetic matrices free from xenogenic elements.	(5, 105, 107)
Inability to mimic tumor stiffness: EHS and collagen matrices do not accurately replicate the stiffness of the tumor ECM.	Develop matrices with varying stiffness to better mimic the mechanical properties of the tumor ECM.	(63, 108–113)
Lack of tunability: EHS matrix and collagen matrices have limited control over biochemical and mechanical properties.	Engineer matrices with adjustable biochemical and mechanical properties to mimic the tumor ECM and patient-specific features.	(5, 63, 107, 112, 114–116)
Viscous nature: EHS matrix poses challenges in scaled pharmaceutical applications due to its viscosity.	Develop less viscous matrices suitable for automated liquid handling and high-throughput applications.	(5)
High cost: EHS matrix is expensive, limiting its use in high-throughput drug screens and clinical settings.	Develop cost-effective alternatives or optimize production methods to reduce expenses.	(5)
Heterogeneous architecture and undefined collagen fibril size in collagen matrix	Optimize gelation conditions to achieve a consistent and defined collagen matrix structure	(117–119)
Lack of patient-specific features: Animal-derived matrices do not capture patient-specific characteristics of the tumor ECM.	Design matrices that can reproduce patient-specific characteristics of the tumor extracellular matrix.	(5)
Lack of scalability: Scaling up the production of the EHS matrix for widespread use raises ethical concerns due to the animal burden.	Develop alternative matrices that can be produced at a larger scale without ethical concerns.	(5, 63, 115, 120, 121)
Synthetic PEG-based hydrogels lack the structural features present in the native ECM.	Design biopolymer-based matrices that offer cellular-scale structural features while maintaining reproducibility and tunability.	(5, 63, 113, 115, 120, 121)

### 4.1 Murine Engelbreth-Holm-Swarm matrix

Solubilized reconstituted basement membrane extracts derived from EHS sarcoma mouse cells are commonly used as substrates for the 3D culture of both healthy and cancer organoids. These extracts are available commercially under various trade names, including Gibco Geltrex, Trevigen Cultrex, and Corning Matrigel (122, 123). EHS matrix contains several ECM proteins, primarily laminin and collagen IV, which remain in the reconstituted matrix after extraction (124). This matrix is widely used in cancer organoid research due to its ability to provide a complex microenvironment containing essential elements such as ECM components, growth factors, and cytokines. This environment supports the survival and growth of various neoplastic and TME cell types. However, due to the EHS matrix being sourced from animals, it is susceptible to batch-to-batch variation and contains ill-defined and xenogenic contaminants that can unpredictably influence organoid phenotype (105). For instance, Matrigel consists of about 14 060 unique peptides and almost 1851 unique proteins, many of which are known to affect the behavior of cancer cells (106). Matrigel also exhibits a lack of consistency in protein content between batches (106). Furthermore, the biochemical and mechanical properties of the EHS matrix cannot be adjusted, making it unsuitable for reproducing patient-specific tumor ECM characteristics. In addition, the EHS matrix cannot accurately mimic the tumor extracellular matrix stiffness, which is usually higher than the tumor and healthy matrix (108, 109). Additionally, its high cost and viscous nature hinder its use in scaled pharmaceutical applications due to challenges with automated liquid handling. Furthermore, the origin of Matrigel from mouse cells poses a hindrance to its application in human clinical transplantation, given the potential for immunogenicity (125). The limitations of EHS matrix make it challenging to investigate cancer cell behavior and hinder its use in high-throughput drug screens and clinical settings. Addressing these limitations would still require a substantial animal burden to scale up production for widespread pharmaceutical use, raising ethical concerns.

### 4.2 Collagen matrix

Solid tumors often exhibit a heightened desmoplastic response which is frequently linked to elevated collagen deposition and reorganization, predominantly types I-IV. This increased collagen content influences various aspects of cancer biology via intricate biochemical and biophysical signaling mechanisms (126). Accordingly, the use of collagen type I matrices as a cost-effective and low immunogenicity biomimetic substitute for the EHS matrix has gained popularity in *in vitro* cancer organoid modeling. Collagen, often obtained from animal sources, poses comparable limitations as EHS matrix, which include variation between batches, restricted tunability in both biochemical and mechanical properties, and contamination with undefined and xenogenic elements (5, 107). Moreover, the microstructure of the collagen hydrogel, including fibril alignment and diameter, is highly influenced by the rate of temperature and pH change during the gelation process (117). Consequently, collagen gelation conducted under variable environmental conditions can result in a heterogeneous collagen fibril size and architecture that can significantly impact cell-matrix interactions (118). Numerous studies have explored techniques for regulating collagen matrix growth factor loading capabilities, mechanical properties, and architecture (117, 119). However, these approaches frequently necessitate the use of potentially toxic substances or involve specialized chemical modifications to the collagen protein. These modifications may affect the native crosslinking and ligand availability.

#### 4.2.1 Standardization of three-dimensional matrices: Synthetic-based hydrogels

Engineered/Synthetic matrices can address the issue of utilizing animal-derived extracellular matrices. These engineered and tunable matrix platforms can provide distinctive insights into the roles of the ECM in modulating tumor organoid phenotype and associated drug response. For instance, Gjorevski et al. discovered that specific characteristics of the 3D synthetic PEG-based matrix were essential for facilitating the initial development of adult mouse Lgr5+ intestinal stem cell colonies, as well as their subsequent transformation into organoids (110). The authors reported that PEG matrices with intermediate stiffness (~1.3 kPa), decorated with the integrin-binding RGD peptide, were best suited to promote stem cell colony formation. This process is mediated through the Hippo pathway/Yes-associated protein and is followed by a transition to a softer PEG hydrogel (~190 Pa) supplemented with full-length laminin to support subsequent intestinal organoid differentiation. In another study, Hernandez-Gordillo et al. developed a synthetic matrix with adjustable biophysical and biomolecular properties that facilitated the human intestinal and endometrial organoid culture from multiple different donors (111). Their findings indicate that low-stiffness (~100 Pa) 8-arm PEG-macromer hydrogels, modified with ECM- and integrin-binding peptides (GFOGER) and crosslinked with matrix metalloprotease (MMP)-degradable peptides, offer efficient organoid formation and proliferation comparable to EHS-matrix controls. Furthermore, Cruz-Acuña et al. demonstrated the feasibility of human intestinal organoid growth from pluripotent stem cells in a fully defined PEG-based synthetic matrix. The matrix consisted of a 4-armed maleimide-terminated poly(ethylene glycol) macromer with tunable polymer density and integrin-binding peptides (114). They indicated organoid differentiation into mature intestinal tissue upon *in vivo* injection and highly reproducible organoid culture using this synthetic ECM. Xiao et al. (112) developed a hybrid engineered brain-mimetic biomaterial matrix using PEG combined with the RGD integrin-binding peptide and crosslinked with hyaluronic acid (HA) (53). The aim was to create an ECM for the GBM organoid model that accurately reproduces the pathophysiological interactions of GBM cells with the distinct features of the brain’s ECM, which is notably enriched in HA. They demonstrated the contribution of specific GBM cell–ECM interactions to the resistance against erlotinib, an EGFR tyrosine kinase inhibitor. Although synthetic PEG-based hydrogels have various advantages for precise tunability of material properties, they are often associated with high swelling and lack of cellular-scale structural features present in the native ECM. Synthetic ECMs may harbor unreacted groups that possess cytotoxic properties (127). Furthermore, their application in medical implants or organoids containing immune components could elicit immunological responses in the body, potentially influencing the outcomes of research in immunotherapy (128).

##### 4.2.1.1 Standardization in primary liver cancer

We have discussed the limitations associated with Matrigel and collagen in organoid culture. To address these issues, numerous research endeavors have aimed to develop chemically defined hydrogels suitable for organoid culture. For instance, Ye et al. (129) introduced an innovative hydrogel using polyisocyanopeptides (PIC) and laminin-111 for liver organoid culture. PICs, synthetic polymers, exhibit thermosensitive properties, rendering them easily manageable and highly promising for clinical applications. They demonstrated that liver organoids cultured in a finely tuned PIC hydrogel experience similar proliferation rates as those seen with Matrigel. Furthermore, the rate of proliferation was influenced by stiffness, where organoid growth was most favorable at lower stiffness levels. In another study, Liu and colleagues (130) designed synthetic supramolecular hydrogels using bis-urea amphiphiles containing lactobionic acid (LBA) and maltobionic acid (MBA) ligands. These hydrogels closely resemble the ECM due to their dynamic and fibrillary structure. These findings highlight the potential of carbohydrate-functionalized hydrogels as platforms for liver tissue engineering.

#### 4.2.2 Standardization of three-dimensional matrices: Biopolymer-based matrices

To overcome animal-derived and PEG-based matrices limitations, scientists have designed biopolymer-based scaffolds for organoid cultures. Biopolymer-based matrices maintain superior homogeneity and reproducibility compared to matrices derived from animal sources. For instance, DiMarco and colleagues created a tunable and chemically well-defined recombinant elastin-like protein (ELP) matrix to investigate how matrix properties and geometric culture configuration influence the formation and growth of primary adult murine organoids (115). The study revealed that reducing the mechanical stiffness to approximately 200 Pa and enhancing cell adhesivity through a high RGD ligand concentration led to increased organoid formation, which was comparable to the collagen matrix controls. The limitless potential of engineering and tuning recombinant matrices allows for improved performance and identification of microenvironmental cues influencing organoid formation, differentiation, and function. However, immune responses may be provoked by particular recombinant proteins and self-assembling peptides (131–133). Simply confirming the human origin of the recombinant protein does not guarantee its lack of immunogenicity (134). Moreover, to mitigate the inclusion of additional immunogenic factors, such as bacterial endotoxin, the expression of proteins for clinical purposes is ideally conducted within mammalian or yeast expression systems. Broguiere et al. proposed using fibrin gels derived from purified human plasma fibrinogen as a well-defined, animal-free matrix with adjustable stiffness and pore size. They demonstrated that fibrin gels supplemented with purified laminin-111 and RGD adhesion domains on the ECM supported the growth and generation of murine and human epithelial organoids from healthy and cancerous tissues (63). As a result, they proposed that this engineered hydrogel could be widely adopted as a well-defined substitute for basement membrane extract in various applications. Hunt et al. have conducted a study in which they employed a hybrid matrix composed of hyaluronan and ELP, to encapsulate, proliferate and differentiate adult human, tissue-derived intestinal organoids in a 3D environment (113). They demonstrate that the interplay of various matrix signaling cues such as integrin-ligand concentration, stress relaxation rate, hyaluronan presence, and matrix stiffness controls the growth rate and formation efficiency of intestinal organoids from single cells. Organoids exhibited comparable growth rates to those of EHS-matrix controls for at least 12 passages during serial culture within designed hydrogels.

Prince et al. (135) proposed a nanofibrillar hydrogel called EKGel, which was utilized for the development and progression of breast cancer patient-derived organoids. The study demonstrated that organoid culture in EKGel exhibited comparable histopathological characteristics, gene expression patterns, and drug responsiveness to both their original tumors and patient-derived organoids grown in basement membrane extract (BME). Moreover, EKGel exhibited advantages such as decreased variability between batches, a wide range of mechanical properties, and reduced contamination from mouse cells. These findings establish EKGel as an enhanced alternative to BME matrices, facilitating the initiation, growth, and maintenance of breast cancer PDOs. In another study, Baker and colleagues conducted a study in which they created a well-defined hydrogel with properties resembling biological systems (116). This hydrogel consisted of hyaluronan and a matrix metalloproteinase-cleavable (MMPx) crosslinker. To ensure the tunability of the resulting HA-MMPx hydrogel, oxime crosslinking was employed. The researchers demonstrated that primary breast cancer cells obtained from patient biopsies formed organoids when cultured within the HA-MMPx hydrogel. These organoids exhibited distinct growth rates and drug response compared to those grown in Matrigel^®^, highlighting the significant influence of the extracellular environment on cell behavior. Notably, the HA-MMPx hydrogel did not induce any bias in the immune cell response when tested *in vivo* and supported the development of diverse organoid phenotypes *in vitro*.

##### 4.2.2.1 Standardization in primary liver cancer

To address the issues associated with Matrigel, Collagen, and synthetic polymers, researchers have attempted to develop defined biopolymers as an ECM. For example, Dong et al. (136) introduced a liver TME simulation platform using a defined alginate-gelatin hydrogel to culture patient-derived tumor organoids. They demonstrated that the obtained organoids accurately recapitulated both the biomechanical and biological properties of the TME. When combined with hepatocyte growth factor (HGF), these organoids could preserve various types of stromal cells. The response of patient-derived tumor organoids to various drugs varied among individuals. This model offers benefits such as ease of use, affordability, a high success rate, rapid generation, and the ability to handle a large number of samples. Willemse et al. (137) introduced a method for culturing human cholangiocyte organoids within hydrogels derived from the liver extracellular matrix (LECM). Their study demonstrated that these hydrogels effectively support the growth of cholangiocyte organoids while preserving their phenotype and gene expression profile. The study suggests that adopting liver ECM hydrogels as a substitute for tumor-derived BME could unlock the full clinical potential of human cholangiocyte organoids. Tienderen et al. (138) cultured cholangiocarcinoma organoids (CCAOs) in native tumor and liver ECM obtained by decellularization. They demonstrated that the transcriptome of CCAOs cultured in tumor-derived ECMs showed more resemblance to that of patient-matched CCA tissue *in vivo*, in contrast to CCAOs grown in BME or liver matrices. Krüger et al. (139) designed a cellulose nanofibril (CNF) hydrogel and examined its potential as a substitute clinical-grade scaffold for liver organoid differentiation. Findings revealed that the CNF hydrogel possesses appropriate mechanical characteristics for differentiation, yielding hepatocyte-like cells with comparable or enhanced functionality compared to Matrigel. Consequently, due to its precisely defined and adjustable chemical composition, the CNF hydrogel emerges as a promising alternative to Matrigel for liver organoid cultures.

## 5 Standardizing the prevention of microbial contamination in organoid culture

Organoid generation can be hindered by microbial contamination (30), particularly in organs such as the colon and rectum, which harbor microbiota (140). Recent research has highlighted the impact of contamination on organoid derivation success rates, with microbial contamination limiting success to 74% (141). To reduce contamination risk, washing steps and antibiotics are commonly used in organoid cultures, but there is no consistent, optimized, and standardized protocol, washing steps, or choice of antibiotics/reagents. Researchers use washing with PBS or antibiotics such as primocin, penicillin/streptomycin (p/s), normocin, gentamicin/amphotericin B, or plasmocin, either individually or in combination, to prevent contamination (30, 142–150). Marinucci et al. (151) attempted to standardize washing protocols and antibiotic use to prevent contamination. They found that non-washed samples had a contamination rate of 62.5%, which decreased to 50% and 25% with PBS or p/s-containing PBS, respectively. Interestingly, none of the organoid cultures washed with PBS/primocin were contaminated (151). Moreover, adding p/s to the washing solution reduced cell viability compared to primocin (151). Establishing easy-to-follow protocols for preventing microbial contamination could improve organoid generation success rates.

## 6 Conclusion

Organoid culture has the potential to revolutionize drug discovery, particularly in the context of complex diseases such as primary liver cancer, where the presence of intratumor and interpatient genetic heterogeneity contributes to the observed lack of targeted agent activity and variability in drug responses. To maximize the potential of organoid culture technologies in preclinical research, personalized medicine and clinical settings, standardization of this model is crucial. By establishing consistent and reproducible protocols for generating organoid models, researchers can ensure reliable results and comparisons between studies. To achieve this standardization and unlock the full potential of cancer organoids, collaborative efforts from clinicians, biologists, and engineers are necessary. One significant bottleneck is the low culture success rate, which necessitates the optimization of culture medium compositions and further investigation into their applicability to different cancer types. The success rates for liver organoid generation are 26% per biopsy and 33% per patient (17), 29% (152), and 50% (69), which are lower compared to other cancers, such as pancreatic (75%-83%) (54) and colorectal cancers (90%) (30). The low success rate in hepatocyte organoid cultures can be attributed to the absence of epithelial stem cell features in hepatocytes, leading to a slower growth rate in culture platforms. Additionally, the limited availability of fresh tissue, obtained through approaches such as small-needle biopsies (153), may contribute to this low success rate. Furthermore, it is noteworthy that liver organoids are predominantly derived from poorly differentiated tumors (16, 17). Addressing these challenges, there is potential for improving success rates through the refining of media recipes (55), the customization of 3D matrices (110), and the implementation of other standardization approaches based on tumor characteristics. These enhancements have the potential to optimize the conditions for hepatocyte organoid cultures, potentially leading to higher success rates in future studies. Additionally, generating pure tumor cultures and incorporating the tumor microenvironment are critical for successful clinical implementation. Overcoming the limitations of organoid cultures and developing novel technologies based on patient-derived samples are highly anticipated. Microfluidic platforms and high-throughput tools have shown promise in diagnostic and drug development purposes, offering alternative approaches to enhance organoid technology. Furthermore, the development of standardized and robust organoid assays with predefined cutoff values for drug response is essential for their clinical use. Another facet that requires consideration in organoid standardization studies is the concurrent standardization of all aspects of organoid culture—a matter that has been overlooked thus far. Future studies, with larger patient cohorts and focused research, are warranted to address the remaining obstacles and fully harness the benefits of tumor organoids in cancer research and clinical applications.

## Author contributions

LG: Conceptualization, Data curation, Investigation, Visualization, Writing – original draft. CL: Data curation, Investigation, Visualization, Writing – original draft. WG: Conceptualization, Investigation, Supervision, Writing – review & editing.
